# Protein degradation technology: a strategic paradigm shift in drug discovery

**DOI:** 10.1186/s13045-021-01146-7

**Published:** 2021-09-06

**Authors:** Haobin Li, Jinyun Dong, Maohua Cai, Zhiyuan Xu, Xiang-Dong Cheng, Jiang-Jiang Qin

**Affiliations:** 1 Zhejiang Provincial Research Center for Upper Gastrointestinal Tract Cancer, The Cancer Hospital of the University of Chinese Academy of Sciences (Zhejiang Cancer Hospital), Hangzhou, 310022 Zhejiang China; 2 Institute of Basic Medicine and Cancer (IBMC), Chinese Academy of Sciences, Hangzhou, 310018 Zhejiang China; 3 School of Pharmaceutical Sciences, Zhejiang Chinese Medical University, Hangzhou, 310053 China

**Keywords:** Protein degradation, Degradation pathway, Degradation technology, PROTAC, Monomeric degraders

## Abstract

Targeting pathogenic proteins with small-molecule inhibitors (SMIs) has become a widely used strategy for treating malignant tumors. However, most intracellular proteins have been proven to be undruggable due to a lack of active sites, leading to a significant challenge in the design and development of SMIs. In recent years, the proteolysis-targeting chimeric technology and related emerging degradation technologies have provided additional approaches for targeting these undruggable proteins. These degradation technologies show a tendency of superiority over SMIs, including the rapid and continuous target consumption as well as the stronger pharmacological effects, being a hot topic in current research. This review mainly focuses on summarizing the development of protein degradation technologies in recent years. Their advantages, potential applications, and limitations are also discussed. We hope this review would shed light on the design, discovery, and clinical application of drugs associated with these degradation technologies.

## Background

Malignant tumors are one of the major threats to human health and rank as the first or second leading cause of death worldwide [1]. The pathogenesis of malignant tumors is related to the mutation and/or overexpression of pathogenic proteins. Therefore, inhibiting the function of pathogenic proteins represents one of the effective strategies for anticancer therapy [2]. In recent years, many small-molecule inhibitors (SMIs) have been developed and achieved certain therapeutic effects [3–5]. The multi-omics analyses of human cancer have identified a variety of therapeutic protein targets. However, most of these target proteins, such as transcription factors and scaffold proteins, lack active binding pockets for SMIs, which extremely limits the design and development of drugs to target these disease-related proteins [6].

Proteolysis-targeting chimeric (PROTAC) technology and other emerging degradation technologies have brought about a paradigm shift in targeting the undruggable proteins. Compared with the traditional inhibitor, the small-molecule degradation agent does not need to continuously expose the binding site of the protein. In addition, most degraders such as PROTACs require a smaller dosage due to their catalytic properties [7–9]. Although PROTAC technology has many unique advantages, potential limitations (e.g., relatively large molecular weight, a specific E3 ligase-related drug resistance, and the restricted ability to degrade protein aggregates and other non-protein molecules [10]) of the degradation technology represented by PROTAC still limit its development to a certain extent. In this review, we first introduced various technologies that achieve selective degradation of target proteins using heterobifunctional small molecules through the proteasome pathway. We also reviewed the lysosomal degradation pathway, a major degradation pathway independent of the proteasome, including the endosome/lysosome pathway and the autophagy pathway [11, 12]. In addition to heterodimeric molecules, the small monomeric compounds that directly promote protein degradation are also discussed here.

### Heterobifunctional molecule-proteasome pathway

The ubiquitin–proteasome system (UPS) is one of the major pathways responsible for degrading proteins to maintain cell homeostasis and participates in the degradation of more than 80% of the protein in cells [13]. This system consists of ubiquitin, proteasome, enzymes, and intracellular proteins or target substrates, playing a key role in a variety of metabolic processes in cells, such as intracellular signal transduction, transcription, and cell cycle regulation [14]. The UPS degrades proteins in a multistep process. The first stage involves the interaction between ubiquitin and substrate protein: one molecule ATP is consumed to activate the ubiquitin molecule to generate E1-ubiquitin complex in the presence of E1 ubiquitin-activating enzyme. Then, the activated ubiquitin is transferred to the E2 ubiquitin-conjugating enzyme to release E1 and forms an E2-ubiquitin complex. Subsequently, the ubiquitin on the E2-ubiquitin complex is transferred to E3 ubiquitin ligase once E3 recognizes and binds to the substrate protein, and the substrate forms an amide bond with ubiquitin through the ε-amino group of lysine. The second stage is the degradation of the substrate by the proteasome: the ubiquitinated protein can be recognized by the cap-like regulatory particles of 26S proteasome, transported to the cylindrical core of 20S, hydrolyzed into oligopeptides by various enzymes, and finally released from the proteasome to degrade the target protein [15, 16]. Therefore, relying on the UPS system to achieve protein degradation is a very feasible strategy. Here are several protein degradation technologies that depend on the UPS system.

### PROTAC

**Fig. 1:**
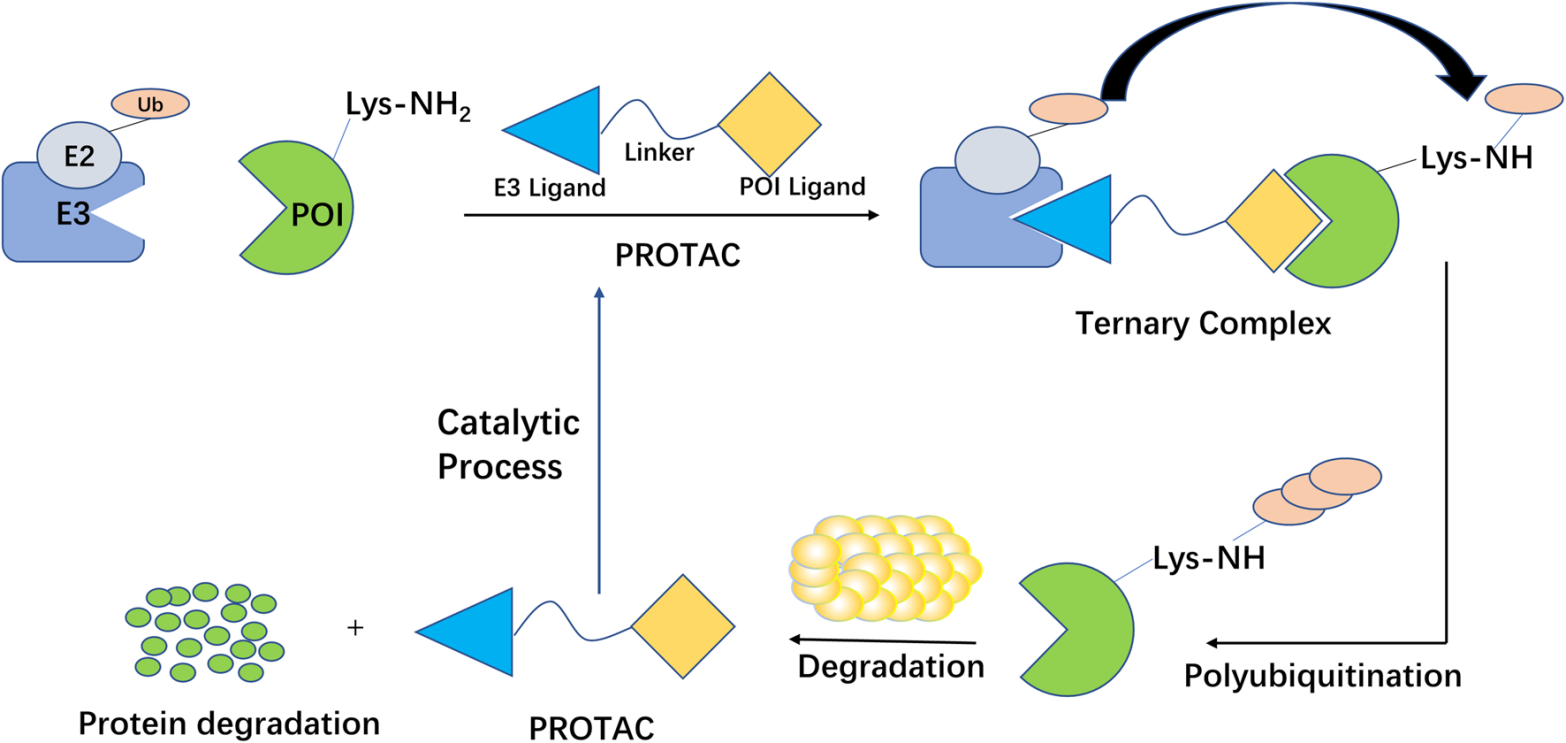
Mechanism of PROTAC to induce protein degradation

PROTAC is now attracting more and more attention because of its great potential in cancer treatment [17]. PROTAC is a heterobifunctional molecule that consists of a ligand of an E3 ubiquitin ligase, a ligand of the target protein, and an intermediate linker [15, 18–22]. The degradation is performed by hijacking the intracellular UPS (Fig. 1). Compared with traditional SMIs, PROTAC overcomes the problem of high doses of SMIs due to its catalytic property and has a stronger sustained efficiency [19]. In addition, PROTAC can degrade the "undruggable" targets, including transcription factors and scaffold proteins [18].

**Fig. 2:**
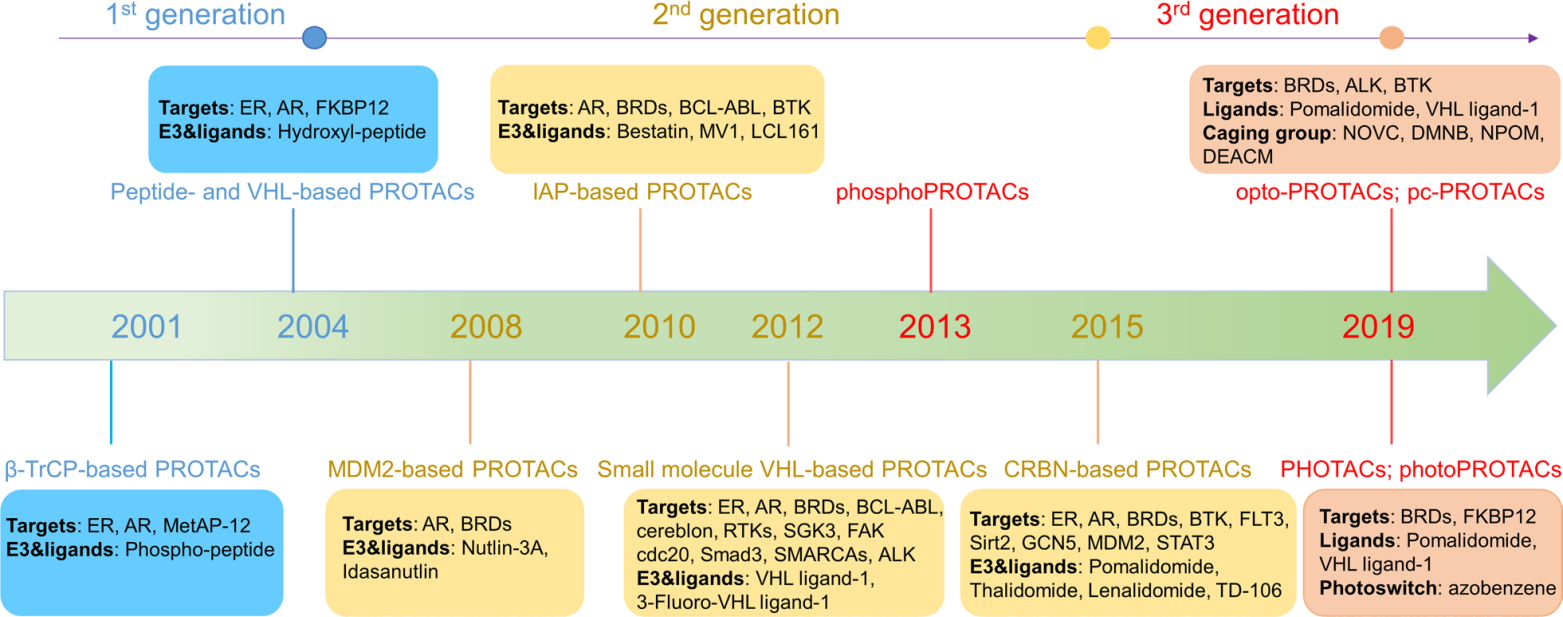
The development process of PROTAC technology

PROTAC has experienced three generations of development (Fig. 2). The first generation of peptide-based PROTAC (called PROTAC-1) was developed by the Crews and Deshaies group in 2001 that applied β-TrCP or VHL as an E3 ligase, but it suffers from poor cell permeability and chemical stability, limiting its clinical applications [18]. The second generation of small molecule-based PROTAC conducted in-depth research on E3 ligase by using MDM2, IAP, VHL, or CRBN as the E3 ligase [15, 18]. Although the degradation efficiency has been greatly improved, there are still problems such as potential off-target specificity, relatively high molecular weight, and toxicity [18, 23, 24]. Further research has developed the third-generation controllable PROTAC, including phosphate-dependent PROTAC (phosphoPROTAC) and light-controlled PROTAC, which can trigger the target protein degradation through activated kinase-signaling clue or visible light, respectively [18, 21, 25, 26]. This method may provide a new approach for PROTAC development. However, given the potential damage of ultraviolet rays to DNA and the inability to penetrate tissues, other spectral regions, such as the near-infrared, have aroused great interest in developing PROTAC [18, 27]. The advantages and disadvantages of each generation of PROTAC are summarized in Table 1.

**Table 1:** A comparison among different generations of PROTACs

	1st generation	2nd generation	3rd generation
Advantages	Larger contact interface with POI and more choices of modification on the drug	Applicable for “undruggable” targets; good cell permeability and solubility	Well-controllable biological characters; causing protein degradation in a highly specific temporal and spatial manner
Disadvantages	Poor cell permeability and stability [18]	Potential off-target effects, relatively high molecular weight, and toxicity [18, 21, 23, 24]	Potential damage to DNA by UV light and low tissue penetration [18, 27]

PROTAC technology is a promising modality to treat diseases, in particular cancer. This is not only reflected in the continuous and rapid depletion of protein of interest (POI) by PROTAC but also in the wider range of potential targets of PROTAC, in particular for "undruggable" targets [18, 25]. Herein, we will highlight some PROTACs that degrade several representative target proteins.

#### Androgen receptor (AR)

**Fig. 3:**
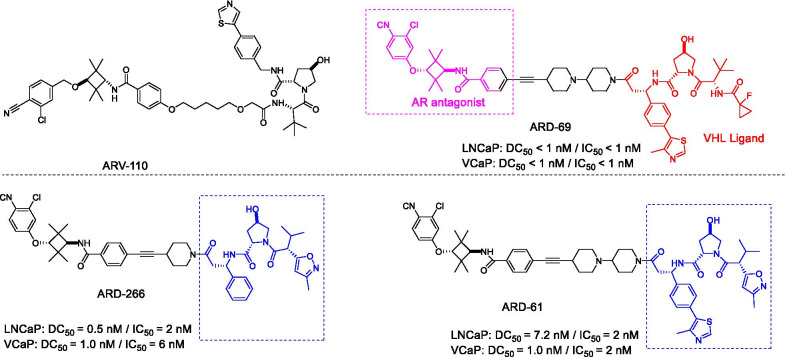
Structures of AR PROTACs

Prostate cancer is an important cause of cancer-related death second only to lung cancer among men in developed countries [28]. Although existing drugs have shown good benefits for patients with advanced prostate cancer, limited efficacy has been observed for metastatic castration-resistant prostate cancer (mCRPC), resulting in a high mortality rate [29]. Studies have shown that AR is a therapeutic target for mCRPC [30]. Although some AR antagonists, such as enzalutamide (ENZ) and apalutamide, have been used to treat mCRPC, patients still develop drug resistance [31]. PROTAC-based AR degraders represent a novel approach for treating prostate cancer. Typically, the first PROTAC drug ARV-110 (Fig. 3) developed by Arvinas is used to treat mCRPC. Compared with AR-targeted drugs, ARV-110 has promising efficacy as a targeted degrading agent of AR in models sensitive to ENZ. ARV-110 showed a comparable ability in decreasing prostate-specific antigen (PSA) at a lower dose. In the ENZ-resistant model, ARV-110 can significantly inhibit tumor growth [32]. In addition, ARV-110 specifically degrades AR ≥ 95% in ENZ-treated and drug-resistant prostate cancer xenograft models [33]. ARV-110 has now entered clinical phase II (NCT03888612), and the initial clinical phase I data demonstrated that it has good oral availability, safety, and tolerability [34].

Encouragingly, many researchers have conducted a large number of studies in the field of AR degradation. Han et al. designed and synthesized a series of AR-PROTAC molecules by using different types of AR antagonists and the ligands of two E3 ligases (cereblon (CRBN)/cullin 4A and VHL/cullin 2) (Fig. 3) [35]. After optimization, it was found that ARD-69 effectively degraded AR with a DC_50_ < 1 nM and a D_max_ > 95% in AR-positive prostate cancer cell lines. In the subsequent studies, they found another compound ARD-266, with an effective AR antagonist and a VHL ligand (weak binding affinity to VHL), also effectively induced AR protein degradation with a low DC_50_ value (0.2–1 nM) in prostate cancer cells [30]. This study proved for the first time that E3 ligands with micromolar binding affinity to the E3 ligase complex can also be used to degrade the target protein successfully. Studies have provided evidence that AR also plays an important role in breast cancer [36]. Zhao et al. have reported a degradant ARD-61 (Fig. 3) that can effectively induce AR degradation in human breast cancer cell lines and xenograft tumor models [37]. In comparison with clinically approved AR antagonists (such as ENZ, etc.), ARD-61 exhibited stronger anti-proliferative and pro-apoptotic effects and attenuated the expression of AR target genes in prostate cancer cells in vivo and in vitro. More importantly, ARD-61 was effective in the ENZ-resistant models [38]. In general, AR has been considered as a promising target for both prostate and breast cancers, and AR degradants have more potential to overcome drug resistance as compared with AR antagonists.

#### Bruton’s tyrosine kinase (BTK)

**Fig. 4:**
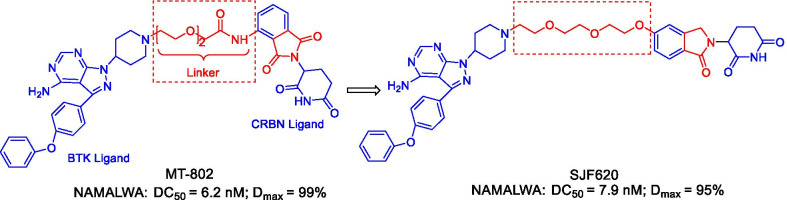
Structures of BTK PROTACs

BTK is a non-receptor tyrosine kinase that is indispensable for the growth, development, and maturation of B cells [39, 40]. Mutations or abnormal signal regulation can cause many diseases related to B-cell malignancies [41]. BTK is also a key regulator of the B cell receptor (BCR) signaling pathway, and it is widely expressed in different types of hematological malignancies. It has been previously reported that irreversible BTK inhibitors (such as ibrutinib) can block its activity by covalently binding to Cys481 in the active site [42, 43]. However, drug resistance caused by the mutation of cysteine to serine at the amino acid 481 (C481S) is the main reason for the interruption of treatment in CLL patients who have been using BTK inhibitors for a long time [44]. Thus, the discovery of new treatment strategies is particularly important, and several BTK PROTACs have been reported [45]. According to available literature, the degradation of BTK can be caused by a reversibly binding PROTAC, while the covalently binding PROTAC inhibited the degradation of BTK [46]. Buhimschi et al. reported that MT-802 (Fig. 4), a PROTAC based on a reversible ibrutinib derivative and the CRBN ligand pomalidomide can effectively induce the degradation of wild-type and C481S mutant BTK at a low nanomolar concentration, along with a degradation rate > 99% [47]. Compared with ibrutinib, MT-802 has a higher selectivity with reduced side effects. Unfortunately, it was found that the pharmacokinetic property of MT-802 was not suitable for in vivo studies, such as a high clearance rate (1662 mL/min/kg) and a short half-life (0.119 h) [43]. As a result, the optimization of MT-802 is needed to improve its pharmacokinetic characteristics. Several compounds were founded to have strong degradation effects and good pharmacokinetic properties by modifying the structure of MT-802. Among them, the most potent degrader SJF620 (Fig. 4), obtained by varying the structure of the CRBN ligand, had a DC_50_ value of 7.9 nM. It can not only effectively induce BTK degradation in NAMALWA cells, but also has better pharmacokinetic properties than MT-802, suggesting its promising potential in the treatment of BTK-related diseases.

#### Focal adhesion kinase (FAK)

**Fig. 5:**
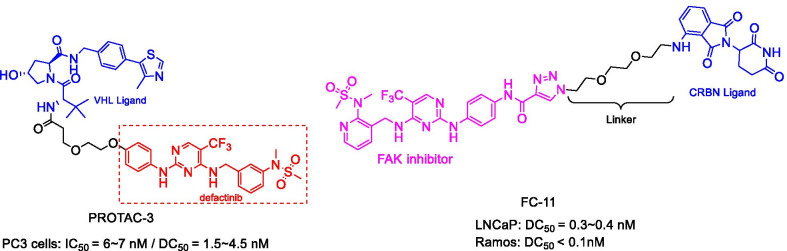
Structures of FAK PROTACs

FAK is a cytoplasmic tyrosine kinase with kinase-dependent enzymatic function and kinase-independent scaffold function, both of which play an important role in the development and reproduction of early embryos [48–50]. However, overexpression of FAK has been detected in a variety of cancers, which provides an important target for immunotherapy [51]. Although various FAK SMIs have been developed, they can only block the enzymatic function of FAK without affecting scaffold protein, finally causing drug resistance [49, 52, 53]. To address this issue, Cromm et al. have developed an effective FAK degradation agent PROTAC-3 (Fig. 5) that can selectively degrade FAK at low nanomolar concentrations [54]. Compared with the FAK inhibitor defactinib, PROTAC-3 showed outstanding inhibitory effects on cell migration and invasion in triple-negative breast cancer (TNBC) cells. Also, Gao et al. developed a PROTAC FC-11 (Fig. 5) by optimizing and characterizing FAK inhibitors and CRBN ligands. The results showed that FC-11 possessed high degradation activity (DR_1 nM_ = 90%, DR_10 nM_ = 99%; DR, the protein degradation relative to DMSO) in the human ovarian cancer cell line PA1 [49]. They observed that many factors affected the degradation activity, including the composition, length, and flexibility of the linkers. Additionally, FC-11 was demonstrated to have the ability to overcome the limitations of SMIs and suppress the non-enzymatic functions of FAK [55].

#### Bromodomain and extraterminal (BET)

**Fig. 6:**
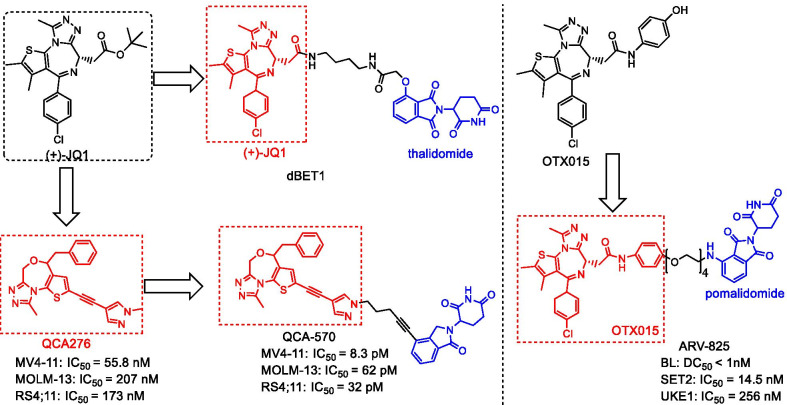
Structures of BET PROTACs

BRD4 is the most distinctive member of the bromodomain and extraterminal (BET) protein family, which has motivated extensive research [56]. Because of its important role in regulating essential oncogenes, BRD4 has become an important target in a variety of cancers, including acute myeloid leukemia (AML) [57], multiple myeloma (MM) [58], and prostate cancer [59, 60]. To date, more than 13 small-molecule BET inhibitors, including JQ-1 and OTX015 (Fig. 6) are in clinical trials for the treatment of cancer and other diseases [61, 62]. The preclinical studies have shown that their IC_50_ values for inhibiting the proliferation of various cancer cell lines range from 100 nM to 1 μM, suggesting their great therapeutic potentials [63, 64]. Considering that many SMIs such as OTX-015 often lead to the accumulation of BRD4 and the incomplete inhibition of cancer cell growth [65], Winter et al. synthesized the first BET-targeting PROTAC dBET1 (Fig. 6) that was composed of the SMI JQ-1 and the CRBN ligand thalidomide through an eight-atom linker N-butyl-2-hydroxyacetamide [58]. BRD4 was significantly depleted in one hour, and it was completely degraded after two hours when a human AML cell line (MV4;11) was treated with 100 nM dBET1. On the contrary, JQ-1 alone is not sufficient to induce the degradation of BRD4 in the AML cell line (MV4; 11). Subsequently, Lu et al. designed and synthesized another BRD4-targeting PROTAC ARV-825 (Fig. 6), composed of OTX015 as the binding part of BRD4 and the CRBN ligand pomalidomide through a PEG linker [56]. In the Burkitt lymphoma (BL) cell line, BRD4 was almost completely degraded (DC_50_ less than 1 nM) in a substoichiometric manner. Compared with SMIs, ARV-825 induced more pronounced apoptosis.

Besides, the other degraders, including ARV-771, BETd-246/BETd-260, and QCA-570, have also been designed and synthesized. ARV-771 has been used in the treatment of CRPC [59], while BETd-246/BETd-260 is more effective than the corresponding BET inhibitor in impairing the growth of TNBC cells [66]. Qin et al. reported a 1,4-oxazepine-based PROTAC QCA-570 (Fig. 6) that can degrade BET protein at low picomolar (pM) concentration in leukemia cells and achieve a complete and lasting tumor regression in mice [67]. Collectively, PROTAC-based BET degraders are more effective than the corresponding BET inhibitors in reducing the expression of BET proteins and decreasing cell growth in preclinical models of solid tumors/hematological malignancies [56, 58, 59, 66–68].

#### Cyclin-dependent kinases (CDKs)

**Fig. 7:**
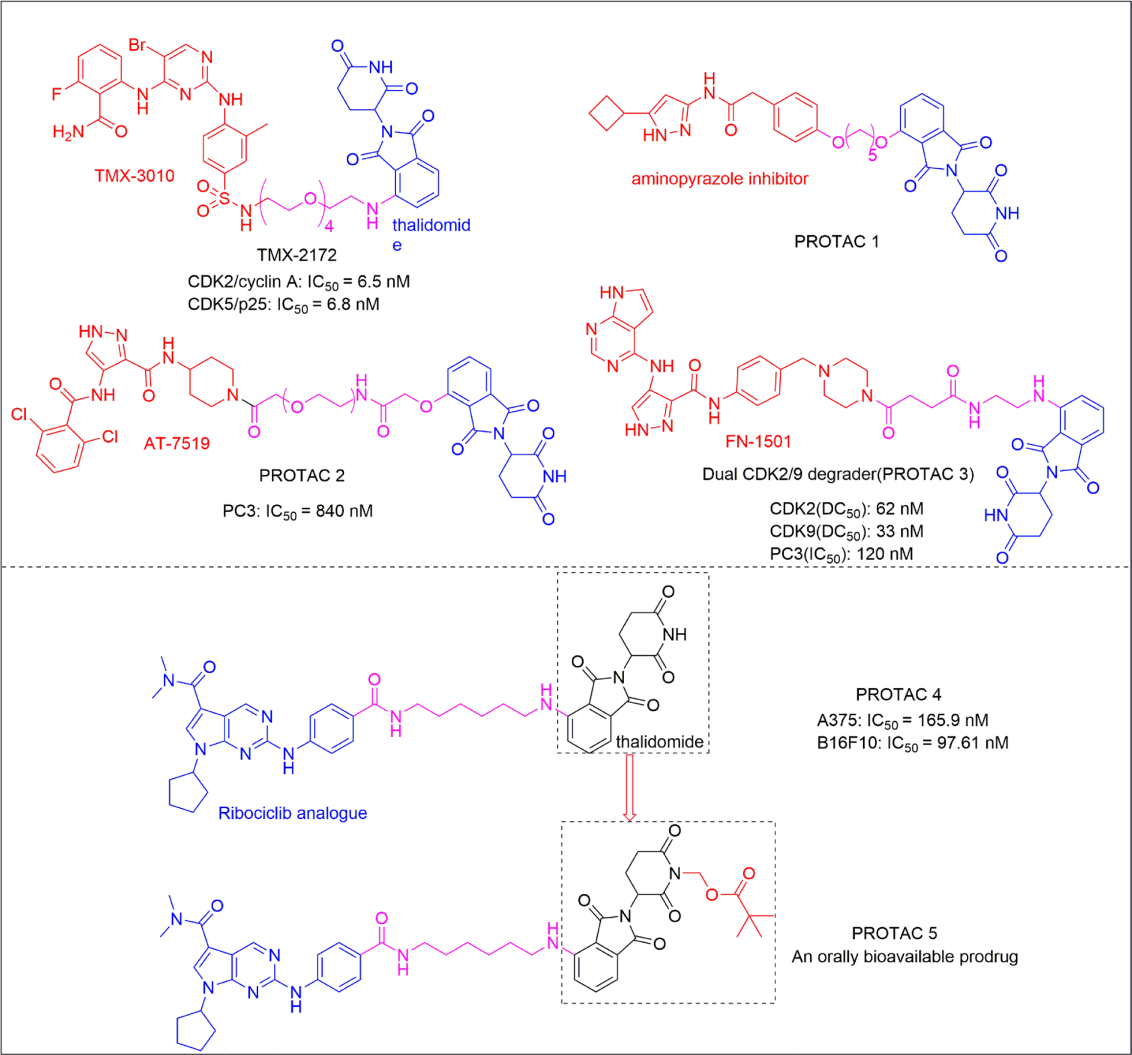
Structures of single- and multi-targeted CDK PROTACs

Cyclin-dependent kinases (CDKs) have been extensively examined as the drug targets for cancer therapy, mainly due to their roles in controlling eukaryotic cell division and proliferation, DNA repair, differentiation as well as apoptosis [69]. Although many CDK inhibitors have shown promising anticancer efficacy, the toxicity caused by the off-targeting effects and the limited selectivity among other CDK homologs often becomes a major clinical problem [70–73]. Interestingly, CDK-targeting PROTACs can specifically degrade homologous proteins to reduce toxicity and improve efficacy [74]. By varying CDK ligands and the CRBN ligand thalidomide, Caroline et al. synthesized PROTAC 1 (Fig. 7) that selectively degraded CDK9 in HCT116 cells without affecting other CDK family members [75]. This is the first example of PROTAC to selectively degrade CDK9. Zhou et al. further reported that PROTAC 2 (Fig. 7) can selectively induce CDK2 degradation at a concentration of 1 µM without affecting CDK5 and CDK9 [74]. They also identified PROTAC 3 (Fig. 7) that can strongly inhibit the proliferation of PC3 cells by simultaneously down-regulating the levels of CDK2/9, thereby achieving a good therapeutic benefit. On this basis, multi-targeted CDK degraders have been further developed. Recently, a CDK2 and CDK5 dual-targeting degrader TMX-2172 (Fig. 7) was identified and showed strong anti-proliferative activity against the ovarian cancer cell line OVCAR by inducing the degradation of CDK2 [76]. Subsequently, Yang's team developed a new type of PROTAC 4 (Fig. 7) that can simultaneously degrade CDK2, CDK4, and CDK6 in melanoma cells [77]. PROTAC 4 can quickly reset the cell cycle and induce apoptosis in various cancer cell lines. Furthermore, researchers have successfully developed an oral bioavailable PROTAC-based prodrug 5 (Fig. 7), which may provide an effective strategy for improving the bioavailability of PROTAC molecules.

### SNIPER

Specific and non-genetic inhibitor of apoptosis protein (IAP)-dependent protein erasers (SNIPERs) are chimeric molecules that are mediated by IAP to induce the ubiquitination and degradation of target proteins through the proteasome pathway. Similar to PROTACs, SNIPERs are composed of an IAP antagonist, the ligand of a target protein, and a connecting linker [78]. However, the difference is that SNIPERs can degrade both the IAP protein (such as cIAP1 and XIAP) and the target protein. Although the specific mechanism is not clear, a recent study has shown that the degradation mechanisms of these two proteins are different [79]. It has been confirmed that the degradation of cIAP1 depends on the interaction between SNIPER and IAP antagonists by developing a series of SNIPERs targeting BRD4, while the degradation of XIAP and BRD4 requires the formation of a ternary complex. Because the overexpression of the IAP family proteins can inhibit the apoptosis of human cancer cells [80], the simultaneous degradation of the target protein and IAP by SNIPERs is beneficial to kill cancer cells [81].

**Fig. 8:**
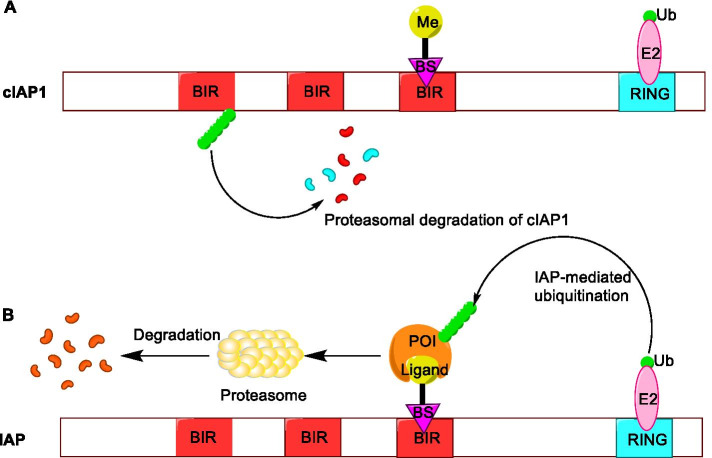
**a** The mechanism of the first generation of SNIPERs technology. **b** Mechanism of IAP-mediated SNIPERs technology inducing target protein ubiquitination and degradation

SNIPERs have undergone two generations of development [82]. In the first generation, SNIPERs are chimeric molecules composed of a target ligand and an IAP ligand bestatin (Fig. 8a). Bestatin, an aminopeptidase inhibitor isolated from actinomycetes, can increase the sensitivity of cancer cells to apoptosis [83]. The initial mechanism of action of SNIPER technology is to use bestatin methyl-ester (MeBS) to bind to the third baculoviral IAP repeat (BIR) domains of cIAP1, and then cIAP1 binds to the proteasome to trigger degradation through self-ubiquitination mediated by the RING finger domain. Later, the methyl residues are replaced by the ligand of the target protein because the methyl residues in MeBS do not participate in the degradation activity (Fig. 8b). In this way, the target protein could be connected to cIAP1-bestatin, ubiquitinated, and degraded by the proteasome [82].

Using this method, a variety of proteins can be degraded, including retinoic acid binding protein (CRABP-II) [84], estrogen receptor-α (ER-α) [85], and AR [86]. Although the first-generation SNIPER compounds could specifically degrade target proteins, they induced the protein degradation only at a concentration of 10 μM or higher. This problem restricts SNIPERs from knocking out proteins in vivo, and thus, it is necessary to develop new SNIPERs with better efficacy.

**Fig. 9:**
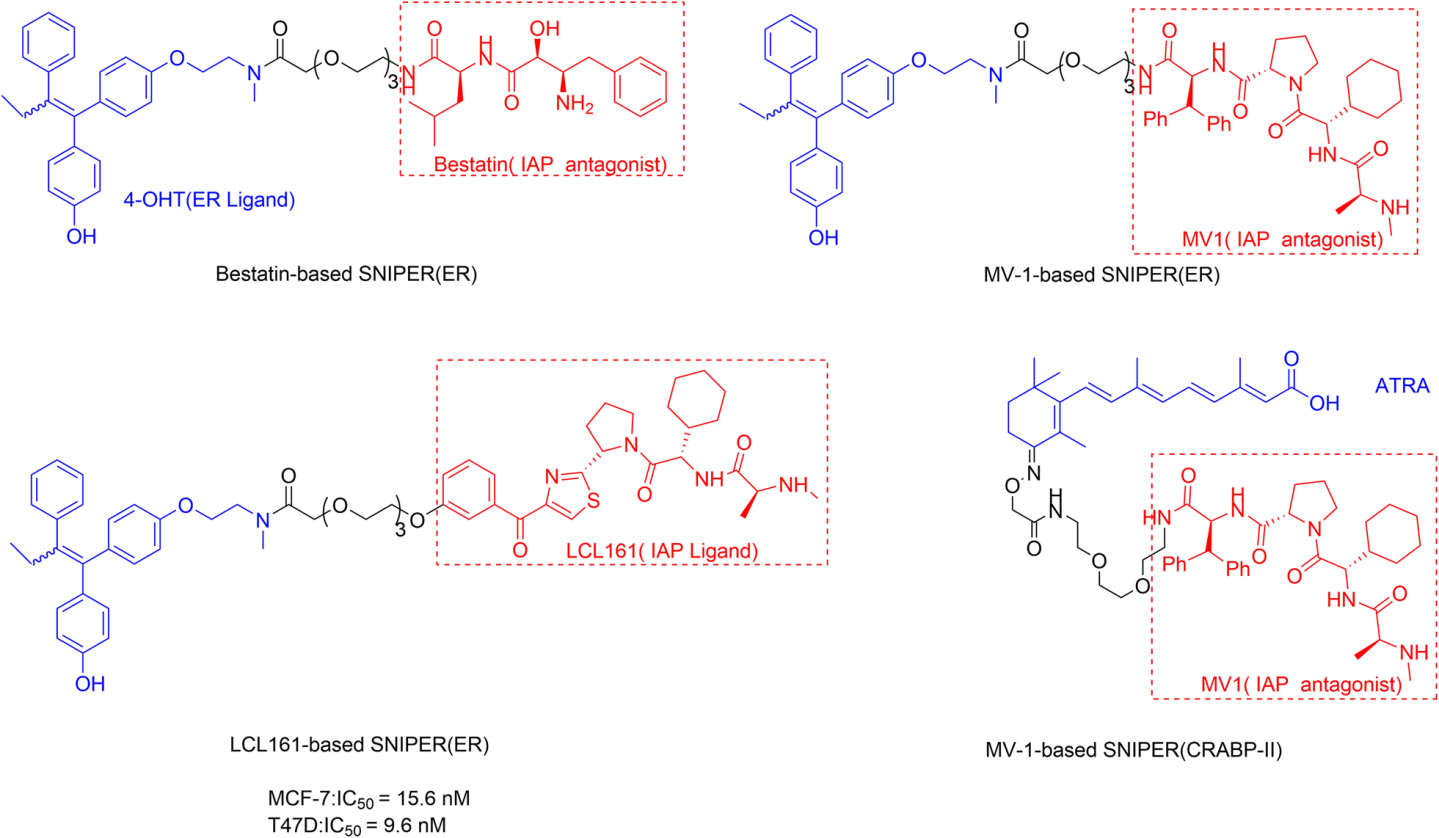
Structures of SNIPERs targeting ERα based on different ligands

The second-generation SNIPERs introduced ligands with binding affinity far higher than bestatin to improve the degradation activity of SNIPERs. SNIPERs composed with IAP antagonist MV1 instead of bestatin as a ligand to connect different target protein ligands can improve the degradation activity of multiple target proteins, including CRABP-II and Erα (Fig. 9) [87, 88]. It was reported that SNIPER (ERα) composed of 4-OHT (ERα ligand) and LCL-161 (Fig. 9) derivative (IAP ligand) with a polyethylene glycol (PEG) linker induced ERα degradation at nanomolar concentration, being 1000 times lower than that of bestatin-based first-generation SNIPER (ERα) [88]. This study has demonstrated the importance of high-affinity IAP inhibitors in the development of SNIPERs.

### HaloPROTAC

**Fig. 10:**
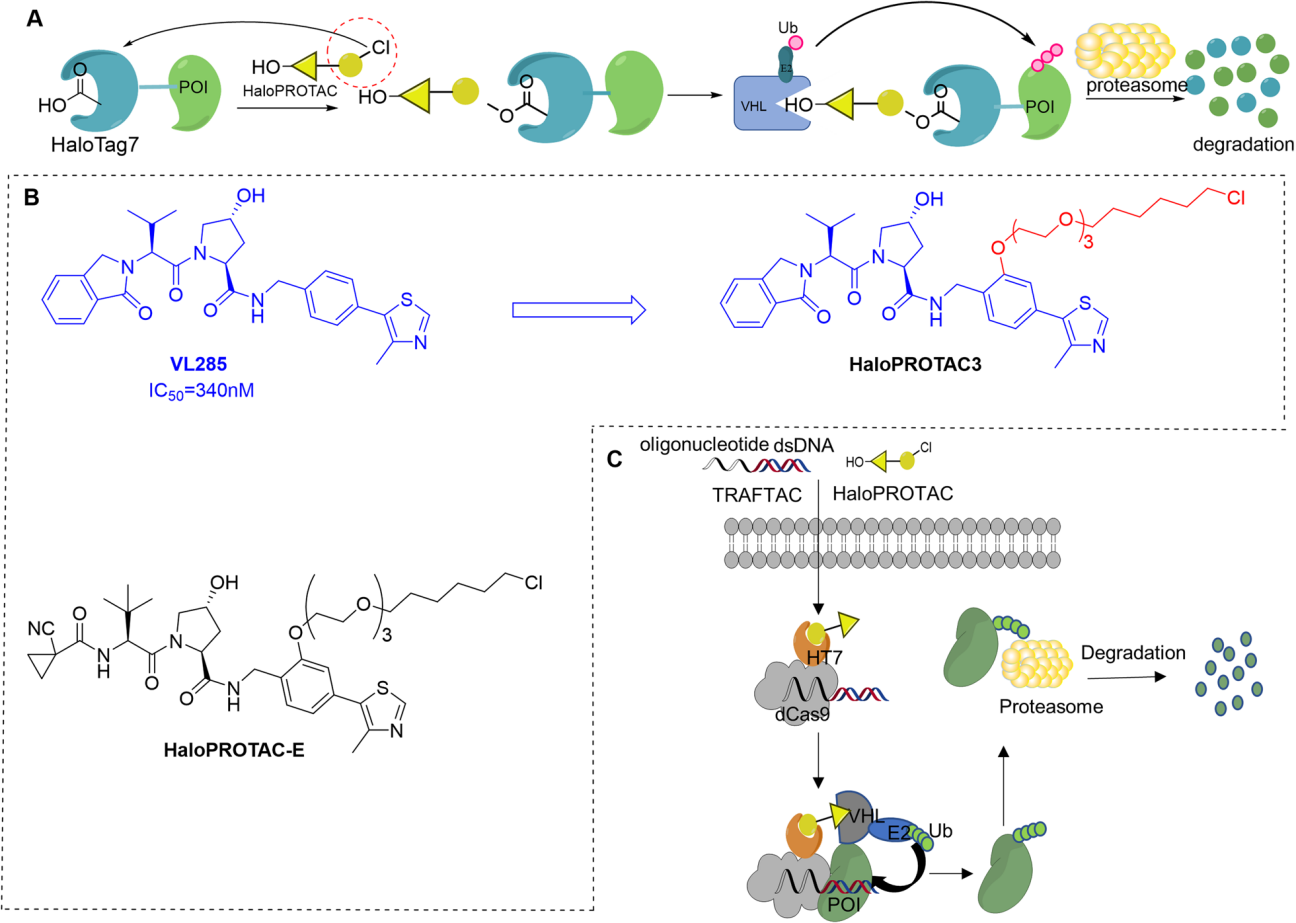
**a** Schematic diagram of the mechanism of HaloPROTAC. In the HaloPROTAC molecule, the yellow triangle connected to the hydroxyl group represents the VHL ligand (hydroxyproline derivative), and the moiety in the red dashed circle represents the chlorinated alkanes. The two parts are connected by a linker. **b** Structures of the VHL ligand VL285 and two effective HaloPROTAC molecules. **c** Schematic diagram of the specific mechanism of TRAFTAC

In 2015, the Crews group developed a novel class of VHL-based PROTACs that can effectively degrade HaloTag7 fusion proteins (HaloPROTAC) [89]. In this study, a chlorinated alkane was connected to the VHL ligand (hydroxyproline derivative) to covalently react with HaloTag, a modified bacterial dehalogenase. In this way, the HaloTag7 fusion protein was brought into proximity with the VHL E3 ligase, and this fusion protein was degraded through the proteasome pathway (Fig. 10a). By varying the length of the linker and VHL ligand, they successfully identified HaloPROTAC3 based on VHL ligand VL285 (Fig. 10b) as the most effective one that degraded GFP-HaloTag protein at 625 nm with a DC_50_ of 19 ± 1 nM. Notably, this method is not only limited to GFP-HaloTag protein but also can be applied to other cytoplasmic proteins such as ERK1 and MEK1. In 2019, the Alessi group expanded the HaloPROTAC technology by combining the HaloPROTAC degradation probe with the CRISPR/Cas9 genome editing technology [90]. They introduced various tags into the endogenous protein and successfully identified HaloPROTAC-E (Fig. 10b) that induced almost a complete degradation of the endogenous HaloTag-fused proteins SGK3 and VPS34 with the DC_50_ values of 3–10 nM and a D_max_ of ~ 95% at 48 h. Interestingly, compared with HaloPROTAC3, HaloPROTAC-E induced greater steady-state degradation of VPS34 with high selectivity. This technology has been widely used in biological research and provides an ideal tool for verifying whether the endogenous target degradation can achieve the expected therapeutic effect. In addition, based on HaloPROTAC technology, the Crews team further developed transcription factor targeting chimeras (TRAFTAC) [91]. Using the ability of transcription factor (TF) to specifically bind to DNA sequences, the dCas9HT7 fusion protein binds to the target TF and E3 ligase simultaneously, and the degradation of the target transcription factor is achieved through the proteasome pathway (Fig. 10c). Moreover, the author also successfully applied this technology to degrade NF-κB and Brachyury transcription factors in this study.

### Hydrophobic tagging (HyT) technology

**Fig. 11:**
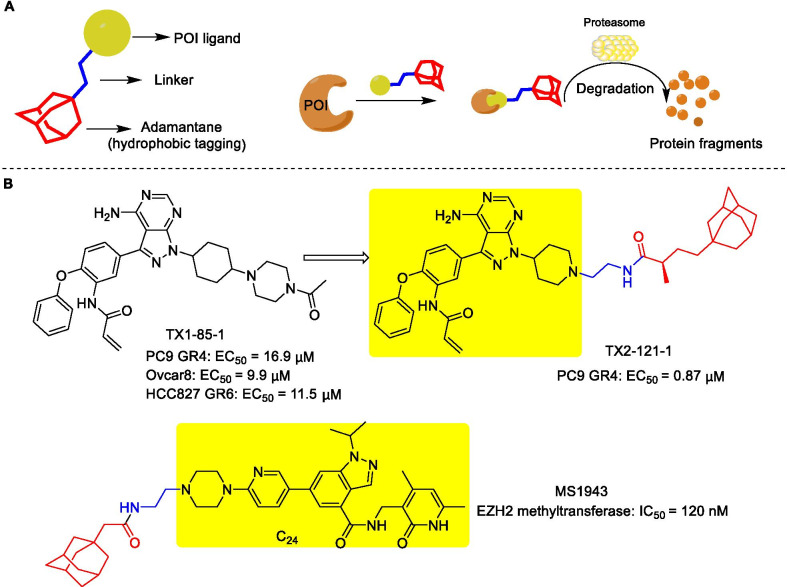
**a** Conceptual diagram of hydrophobic tags and the degradation mechanism. **b** Chemical structures of TX1-82-1, Her3 degrader TX2-121-1, and EZH2 degrader MS1943

Hydrophobic tagging (HyT) technology depends on a bivalent compound containing a ligand of the target protein and a large hydrophobic group to increase the hydrophobicity of the target protein surface, which induces target protein unstable and misfolded, thereby leading to its degradation by the proteasome without E3 ligases or ubiquitination (Fig. 11a) [92]. The HyT technology-mediated degradation starts from the endoplasmic reticulum, and the discovery of the ER degrader Fulvestrant clarifies the concept of hydrophobic tagging for the first time [93, 94]. After that, Crews and colleagues reported a system based on the covalent attachment of a hydrophobic tag to a dehalogenase fusion protein to achieve degradation by the proteasome [95]. Long et al. also proposed a similar concept by using a small-molecule degrader of dihydrofolate reductase (DHFR) composed of arginine (Boc_3_Arg) protected by the hydrophobic group tert-butyl carbamate linked to a non-covalent binding ligand of DHFR trimethoprim (TMP) [93]. Subsequently, Xie et al. synthesized a compound TX2-121-1 (Fig. 11b) by linking TX1-85-1 (a selective Her3 ligand) to hydrophobic adamantane moiety [96]. It enhanced the inhibition of Her3-dependent signals and induced preferential death of Her3-dependent cell lines, with EC_50_ values in the range of 0.8–1.4 μM. Ma et al. identified a selective EZH2 degrader MS1943 (Fig. 11b) based on the highly selective EZH2 inhibitor C24, which not only effectively reduced the intracellular EZH2 level but also selectively killed TNBC cell lines without affecting the normal cells [92]. In addition, the degradation mediated by hydrophobic tags is speculated to be related to the molecular chaperones, which help refold the misfolded protein, masking its exposed hydrophobic and non-polar regions [95, 97]. In a study carried out by Gray et al., the combination of molecular chaperone inhibitors, such as 116-9e or 17-AAG with TX2-121-1 led to an increased Her3 degradation [96]. Generally, this technology is of great significance for targeting druggable and non-druggable proteins.

### Heterobifunctional molecule-lysosomal pathway

Although the proteasome pathway has shown great potential for degrading target proteins, the proteasome-mediated technology (PROTAC [58] and SNIPERs [82], etc.) is usually limited to target proteins containing cytoplasmic domains. However, extracellular proteins and membrane-associated proteins are also the key regulators of physiological and pathophysiological processes such as aging and cancer [98, 99]. Therefore, the selective degradation of these proteins is also of great importance. Contrary to the proteasome pathway, the lysosomal pathway is not limited to degrade cytoplasmic domain proteins [100]. The lysosomal pathway has two mechanisms to degrade target protein, including the endosomal/lysosomal pathway and the autophagy pathway. The endosomal/lysosomal pathway involves a series of membrane-bound intracellular compartments, in which the internalized material and redundant cellular components pass through the early endosomes, endosomal carrier vesicles, late endosomes, and lysosomes for subsequent hydrolysis [101]. The autophagy pathway is to surround the cytoplasm and organelles of the cell through a single isolation membrane, and the edges of the membrane vesicles merge to form a closed double-membrane structure, called autophagosomes. Finally, the autophagosome fuses with a lysosome to become autolysosome, which is degraded in the presence of lysosomal hydrolase [102]. Therefore, both the endosomal/lysosomal pathway and the autophagy pathway can mediate the degradation of target proteins. The recent development of lysosome-mediated degradation technology is discussed more fully below.

### Lysosome-targeting chimera (LYTAC)

**Fig. 12:**
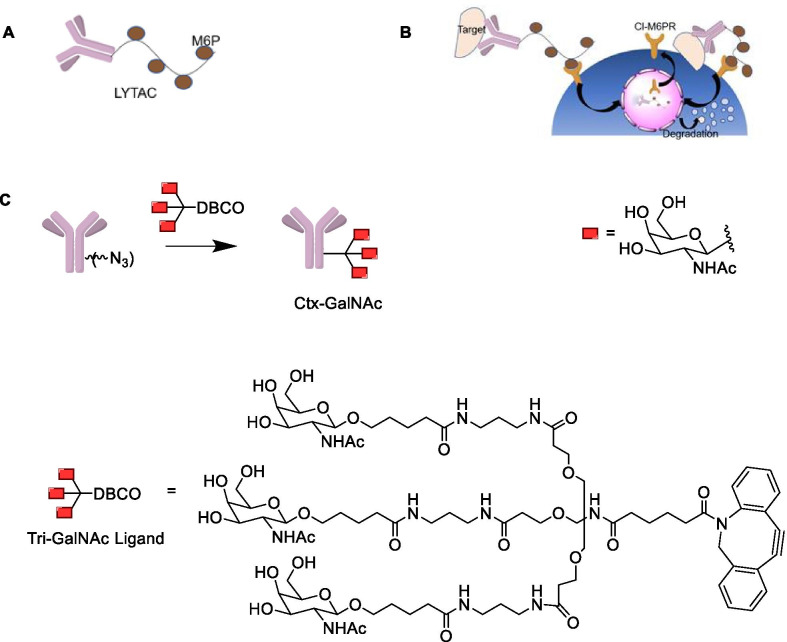
**a** Structure of LYTAC, antibody-conjugated glycopeptide ligand. **b** The mechanism of LYTAC-induced degradation of intracellular proteins and membrane-associated proteins. **c** Antibody-tri-GalNAc conjugate (GalNAc-LYTAC)

Different from PROTAC, LYTAC technology can degrade extracellular proteins and membrane-related proteins through the endosomal/lysosomal pathway. LYTAC molecule is composed of a specific POI antibody or a small-molecule ligand conjugated with a chemically synthesized glycopeptide ligand (Fig. 12a), such as mannose-6-phosphate (M6P), which binds to the cation-independent M6P receptor (CI-M6PR) [103]. CI-M6PR is a lysosomal transport receptor that can effectively deliver proteins to lysosomes for targeted degradation (Fig. 12b). The latest research has conducted an in-depth discussion on the degradation of extramembrane and membrane-related proteins by the lysosomal pathway. Banik et al. proved the feasibility of using LYTAC technology to degrade extracellular proteins by lysosomes, demonstrating that knocking out IGF2R (encoding CIM6PR) will decrease the degradation efficacy of lysosomes [104]. This finding also revealed the importance of the exocyst complex in the LYTAC pathway. To verify whether LYTAC can accelerate the degradation of membrane-bound extracellular proteins, they found that cetuximab (Ctx) conjugating with M6Pn glycopeptide showed substantial degradation of EGFR in HeLa cells (more than 70%). It is interesting to note that the length of the M6Pn glycopeptide has no significant effect on degradation.

To demonstrate whether LYTACs can degrade a given lysosome-targeting receptor expressed by a specific cell type, Ahn and colleagues recently developed a GalNAc-LYTAC that conjugates a cell surface protein antibody-based binding agent to a triantenerrary N-acetylgalactosamine (tri-GalNAc) motif [105]. The degradation mechanism is related to the asialoglycoprotein receptor (ASGPR), a liver-specific lysosome-targeting receptor. They coupled Ctx to a tri-GalNAc ligand and found that the Ctx-GalNAc complex (Fig. 12c) degraded 70% of the cell surface EGFR in Hep3B cells, with the degradation efficacy similar to that of M6Pn-LYTAC. Moreover, it also showed considerable degradation efficacy in HepG2 and Huh7 cells.

### Autophagy-targeting chimera (AUTAC)

**Fig. 13:**
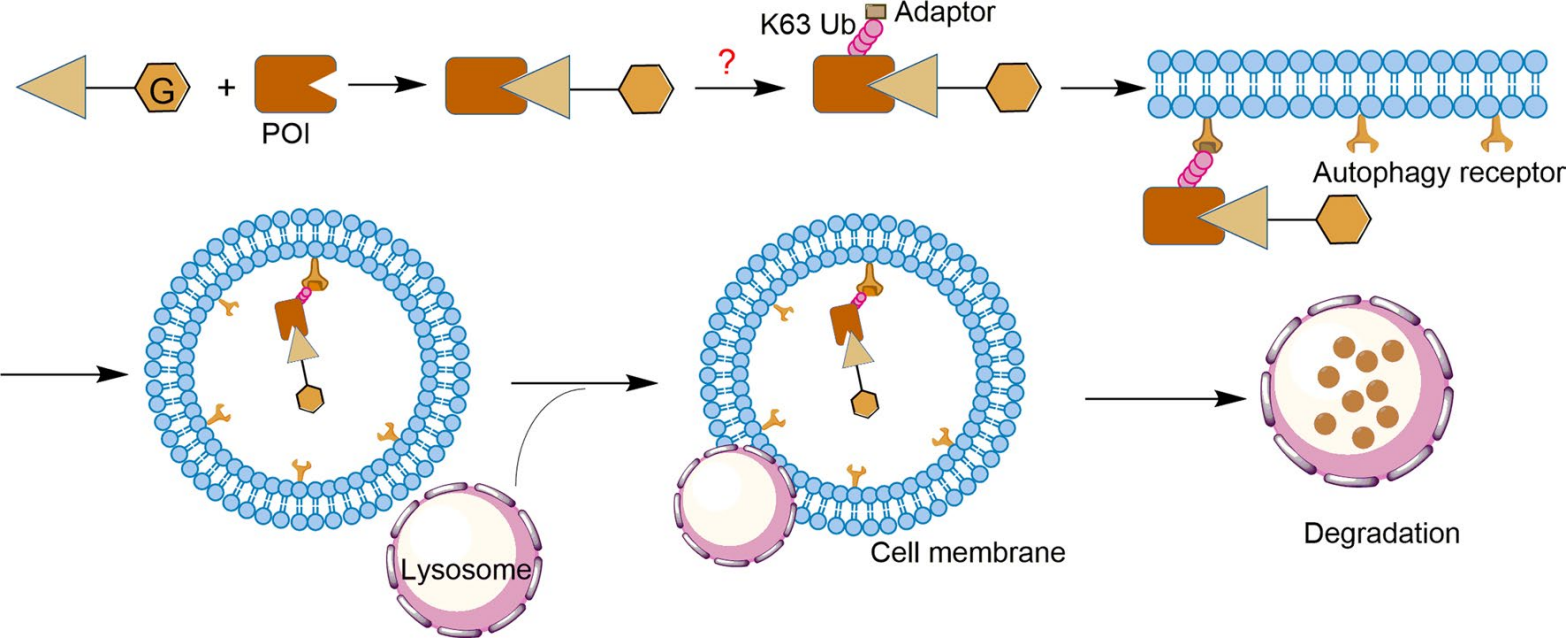
The mechanism of AUTAC technology. The POI ligand in the AUTAC molecular composition is responsible for target specificity, while the degradation tag induces POI to trigger K63 polyubiquitination, which is then recognized by the autophagy receptor SQSTM1/p62 on the cell membrane to form autophagosomes, and finally, selective autophagy degradation is achieved in the lysosome. However, the specific mechanism by which degradation tags trigger K63 polyubiquitination is unclear

Many cell contents such as damaged organelles and protein aggregates are not substrates for proteasome, so they are beyond the scope of targeted protein degradation (TPD) technology like PROTAC [106]. To circumvent this limitation, AUTAC technology has recently been developed to selectively degrade intracellular proteins and intracellular debris through the autophagy pathway. As shown in Fig. 13, an AUTAC molecule contains a degradation tag (mostly guanine derivatives) and a warhead [107]. The cell contents are sequestered into autophagosomes to fuse with lysosomes to achieve degradation in the presence of lysosomal hydrolase [108]. Unfortunately, the current research lacks key information on the mechanism of AUTAC technology. For example, the type of ubiquitin modification to mediate target autophagy cargo is still unclear (Fig. 13) [107, 109]. However, a study on cytoplasmic group A streptococci (GAS) showed that the degradation tag mimics S-guanylationcan to induce K63 polyubiquitination, labeling the substrate protein for selective autophagy [110, 111]. Different from the degradation mechanism by which PROTAC triggers K48 polyubiquitination, the AUTAC degradation mechanism may appear more complicated [107, 109].

**Fig. 14:**
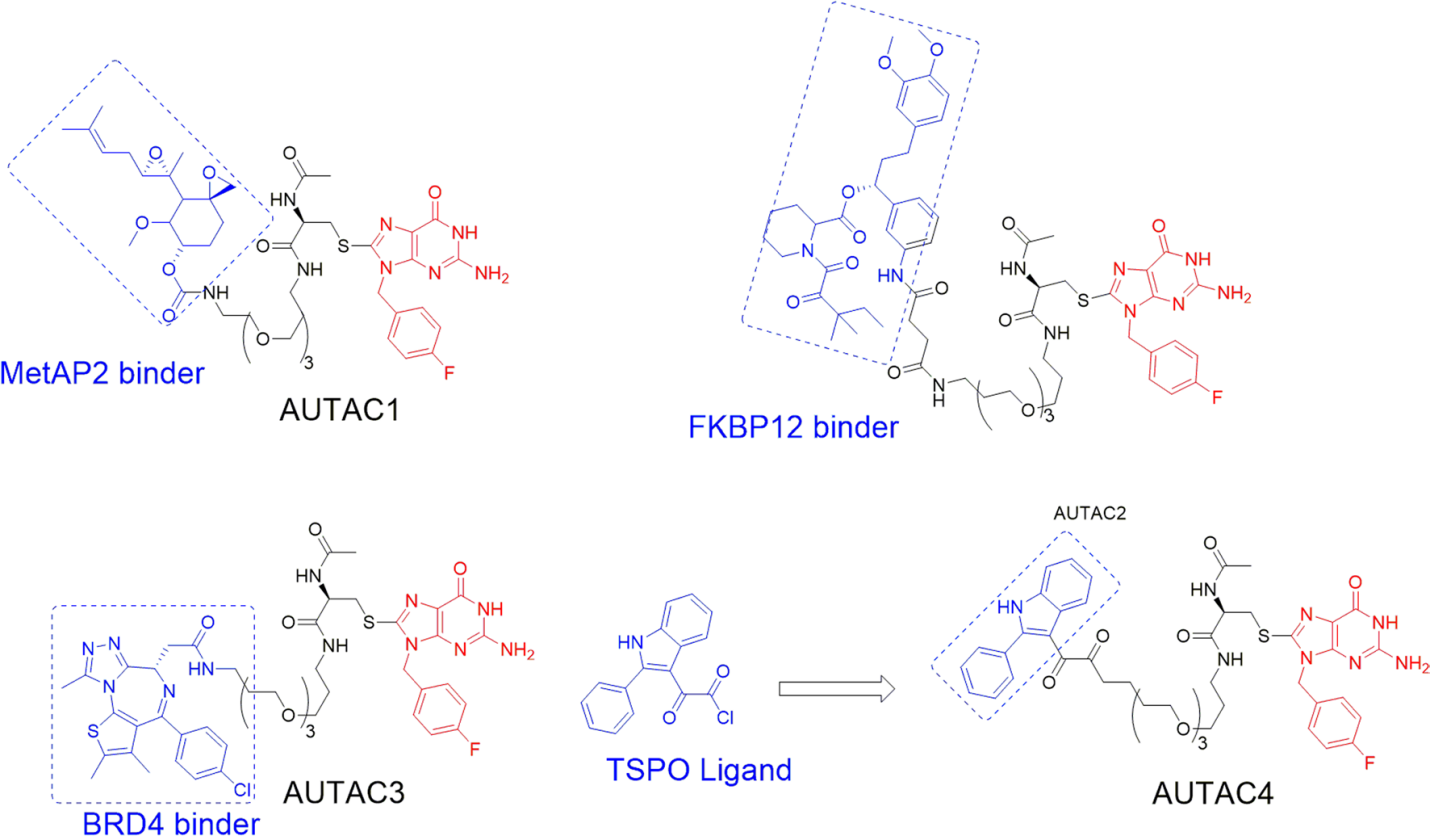
Structures of AUTACs targeting MetAP2, FKBP12, BET, and TSPO proteins

A recent study has reported that AUTAC molecules can promote the phagocytosis of broken or impaired mitochondria, which are related to diseases of aging [112]. The researchers discovered the chimeric molecule AUTAC4 (Fig. 14) that can deliver the guanine tag to the mitochondrial membrane, thereby inducing the polyubiquitination of K63 to achieve selective autophagy. Therefore, AUTAC4 has great potential to improve the activity of mitochondria in the fibroblasts of patients with down syndrome and restore cell function [107, 112].

Currently, AUTAC technology has been successfully applied to degrade many proteins such as MetAP2, FKBP12, BET, and TSPO (Fig. 14). Further studies have shown that mitochondria degradation will induce the degradation of more pathogenic proteins. However, there are still many problems that need to be resolved, including the degradation mechanism of this technology, the factors affecting degradation efficiency, and potential off-target effects [109]. In addition, it seems necessary to develop a method to degrade aggregated proteins using this technology. Although the potential of AUTAC technology has not yet been fully developed, it is foreseeable that AUTAC molecule specifically degrades intracellular proteins or disrupts organelles through selective autophagy, which is expected to become one of the most promising strategies for the treatment of diseases related to organ damage.

### Autophagosome-tethering compounds (ATTEC)

**Fig. 15:**
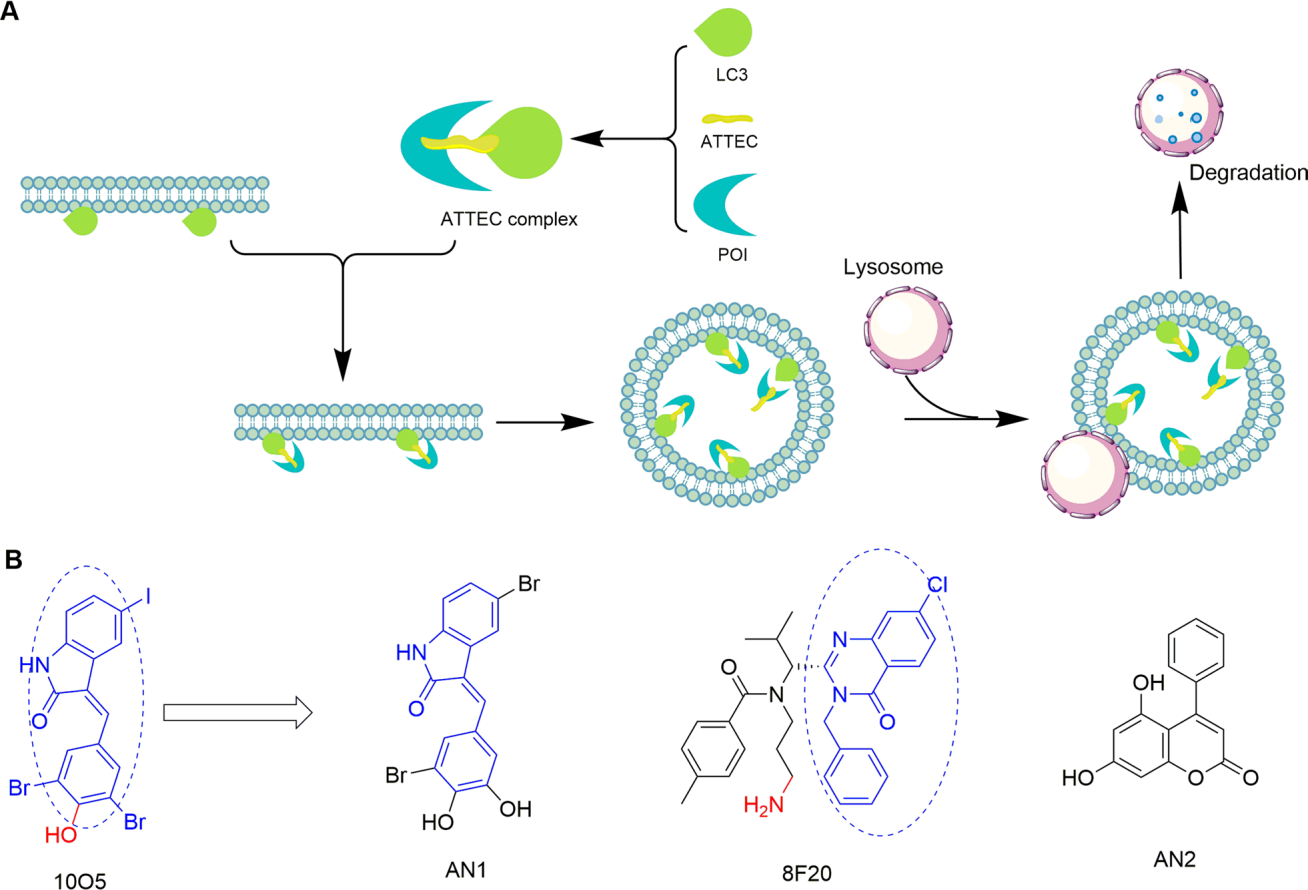
**a** The mechanism of action of ATTEC molecules. The ATTEC molecule interacts with the LC3 protein, and the formed complex is isolated in the cell membrane to form autophagosomes and finally undergo autophagic degradation in the lysosomes. **b** The structures of two hit compounds 10O5 and 8F20 and other identified effective linker compounds AN1 and AN2. The dotted ovals represent the chemical groups that may have protein-compound interactions during screening, and the red groups indicate the SMM label used for the nucleophile-isocyanate reaction

ATTEC is a new degradation technology that has been developed based on the autophagy pathway [113]. However, unlike AUTAC and PROTAC that contain two ligands connected by a linker, an ATTEC molecule lacks a linker, which is similar to "molecular glue" [114]. Compared with AUTAC, the ATTEC molecules exert their functions without ubiquitination process. The mechanism is dependent on such a small molecule that can connect the autophagosome protein LC3 on the surface of the autophagosome membrane with the target protein, thereby triggering the degrading event through the autophagy pathway (Fig. 15a) [113, 115]. At present, ATTEC technology is mainly used to treat Huntington's disease (HD), a neurodegenerative disease caused by mutated huntingtin (mHTT) and extended polyglutamine (polyQ) stretches [116]. Reducing the level of HTT protein has been proven to be a reasonable treatment strategy [117]. Using the small-molecule-microarray (SMM) based on the nucleophile-isocyanate reaction, Ding’s team identified four compounds (Fig. 15b) that interact with LC3 and mHTT without interacting with the wild-type HTT protein [113]. These mHTT-LC3 linker compounds can reduce the level of mHTT in HD cells at nanomolar concentrations and in HD mouse models at a dose of 0.5 mg/kg by intraperitoneal injection. Further experiments also proved that the ATTEC molecule targets autophagosomes for inducing degradation without affecting autophagy [113]. Besides, ATTEC technology has been used to decrease the level of spinocerebellar ataxia type 3 protein (ATXN3) [113].

ATTEC technology provides an ideal method for the treatment of diseases related to polyQ extension lesions. The relatively low molecular weight allows some ATTEC molecules to penetrate the blood–brain barrier, and the potential targets include intracellular proteins and non-protein autophagy substrates. However, there is a lack of principles for the design of chimeras, and little is known about the chemical groups that may interact with LC3 protein [109]. Therefore, it is urgent to clarify the interface structure between linker compounds and LC3, which could provide important information for the design of ATTEC to degrade other proteins.

## Heterobifunctional molecule-ribonuclease pathway

### Ribonuclease targeting chimera (RIBOTAC)

**Fig. 16:**
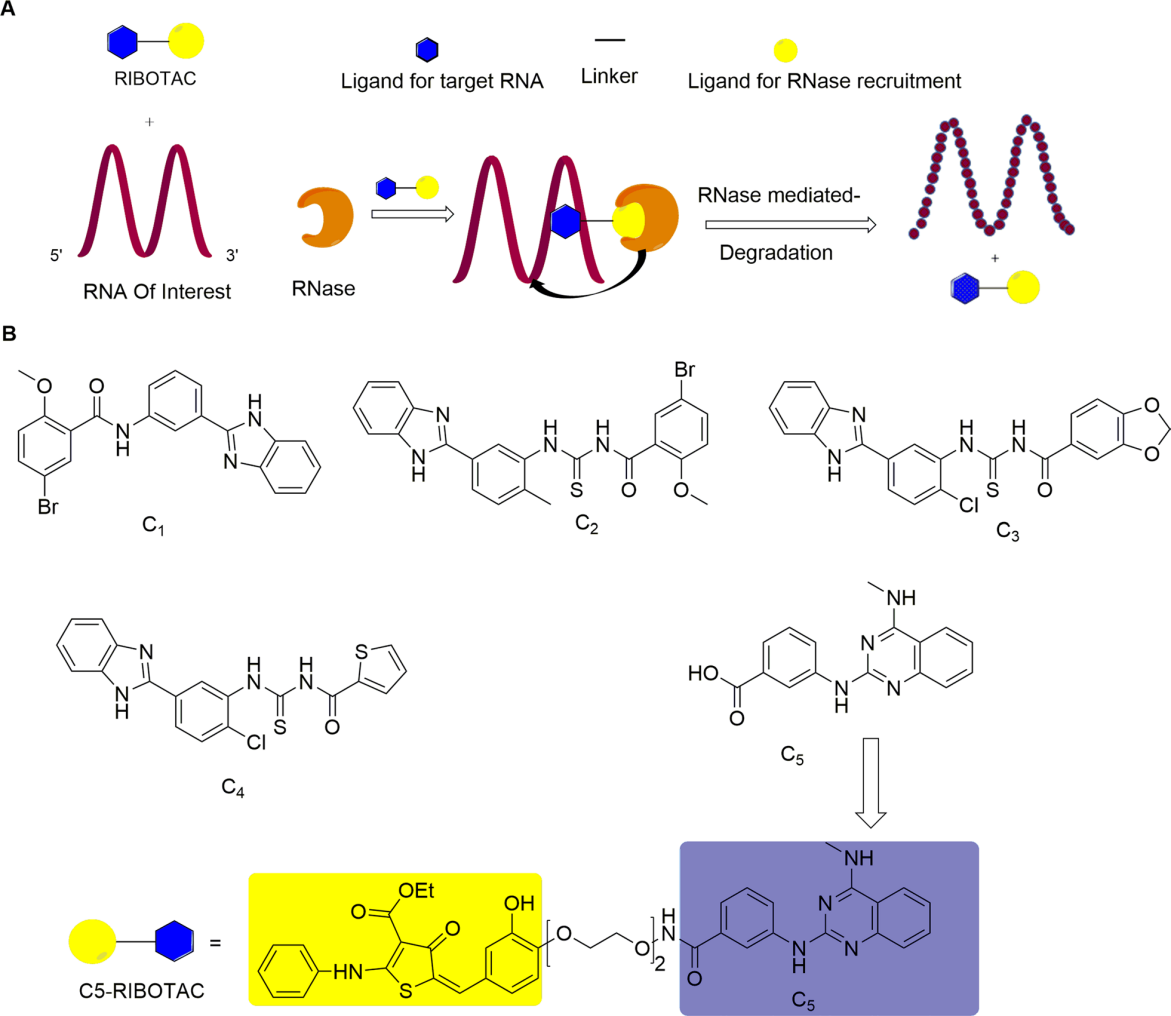
**a** RIBITOC molecule composition and mechanism diagram. **b** The structures of C1-C5 and C5-RIBOTAC

Non-coding RNA (ncRNA), including microRNA, intron RNA, and lncRNA, has a wide range of potential to control gene expression. The mutations and disorders of these RNAs are related to many diseases, such as cancer [118] and neurodegenerative diseases [119]. Thereby, these RNAs have been considered as potential drug targets [120]. The traditional method of RNA degradation is to use antisense oligonucleotides (ASOs) and small interfering RNA (siRNA) [121, 122]. However, poor cell uptake, low tissue-specific transmission (except liver and kidney), and toxicity limit their applicability for disease treatment [123]. The emergence of ribonuclease targeting chimera (RIBOTAC) represents a promising strategy for RNA degradation [124]. This chimeric molecule is similar to the structure of the PROTAC molecule. It is formed by connecting an RNA binding module and a ribonuclease (RNase) recruitment module through a linker. Upon the RNA-binding module binding to the target RNA, RIBOTAC recruits the RNase to promote its degradation (Fig. 16a) [125]. One advantage of RIBOTAC over oligonucleotide-based therapy is its high catalytic property, which can trigger RNA degrading effects at low concentrations. Disney’s team selected five small molecules (C1–C5) (Fig. 16b) from an RNA-focused library (n = 3271) through a microarray screen. These small molecules bind with the model of the SARS-CoV-2 attenuator hairpin (one of the revised models of the SARS-CoV-2 frameshift element) in a dose-dependent manner. Among them, a drug-like small molecule C5 is tightly integrated with the attenuator hairpin model with a *K*_*d*_ of 11 nM. Based on the C5 compound, C5-RIBOTAC (Fig. 16b) was synthesized to recruit ribonuclease to destroy the viral genome [126]. This molecule can selectively induce SARS-CoV-2 RNA degradation and attenuate viral activity by recruiting ribonuclease, showing a great potential to treat the global epidemic of new coronary pneumonia (COVID-19). However, it is a challenge for this technology to discover such a small molecule that can selectively bind to the target RNA [125].

The development of degradation technologies has provided many feasible strategies for the treatment of related diseases. In Table 2, we summarize the above-mentioned technologies in terms of the target range, degradation pathways, advantages, and potential problems.

**Table 2:** Summary of protein degradation technologies

Degradation technology	Target range	Degradation pathway	Advantages	Potential problems	Refs.
PROTAC	Intracellular protein	Proteasome pathway	Targeted degradation of undruggable proteins Specificity Acceptable oral bioavailability (such as ARV-110 and ARV-471) Clear degradation mechanism High degradation efficacy	Large molecular weight Poor oral bioavailability and other pharmacokinetic properties Limited target range Limited available E3 ligase	[21, 127–129]
SNIPER	Intracellular proteins, cIAP1, and XIAP	Proteasome pathway	Simultaneous degradation of target protein and IAP, killing cancer cells that rely on IAP for survival High specificity Sufficient membrane permeability	Need an IAP ligand with high binding affinity The degradation mechanism of cIAP1 and XIAP by the SNIPERs is not well understood	[79, 82, 88]
HaloPROTAC	Endosomal proteins and HaloTag fusion protein	Proteasome pathway	Selectively induce target protein degradation; Improved drug-like properties	The stoichiometric ratio of the chemical components to the protein needs to be labeled The ability to knock the degradation label into the target protein needs to be improved The Halo label itself may become the main target of ubiquitination and degradation	[89, 90, 130]
HyT	Druggable or non-druggable proteins	Proteasome pathway	Some hydrophobic tags are independent of E3 ligases and ubiquitination Wide range of potential targets Universality	High affinity for the target protein ligand The exact mechanism of action remains unclear Potential perturbation of the unfolded protein response pathway may cause off-target effects	[96, 130–132]
LYTAC	Extracellular and membrane-associated proteins	Endosome/lysosome pathway	Degradation does not depend on the UPS system Degrade extra-membrane and membrane-related proteins High controllability	Relative molecular mass is too large There are few types of applicable shuttle receptors Antibody may induce immune response Non-catalytic, low degradation efficiency	[103, 104]
AUTAC	Intracellular proteins and damaged organelles	Selective autophagy pathway	A wide range of potential targets, including damaged organelles such as mitochondria; Proteasome-independent	Lack key information such as the specific molecular mechanism of K63 ubiquitination that mediates S-guanylation to trigger autophagy, as well as its efficiency and potential off-target effects Possible influence on selective autophagy	[107, 109, 112]
ATTEC	Cytoplasmic proteins and non-protein autophagy substrates	Macro-autophagy pathway	The relatively low molecular mass enables it to penetrate the blood–brain barrier A wide range of potential targets Mechanism of direct degradation	Lack of research on designing chimeras Urgent need to clarify the chemical structure of the compound-protein interface	[113, 115]
RIBOTAC	RNA	Ribonuclease pathway	It can degrade RNA at a low concentration	Difficulties in finding small molecules that can selectively bind to the target RNA	[125]

### Monomeric degraders

Although the use of heterobifunctional small molecules to degrade target proteins are attracting more and more attention owing to their promising application prospects, these technologies are still at an early stage and have some shortcomings (as mentioned above). Therefore, the use of these technologies is restricted in some cases. Interestingly, some small-molecule monomeric compounds have been identified as degradants. Although their mechanisms of action are different, they can bind to the target protein and lead to subsequent degradation.

### Molecular glues

**Fig. 17:**
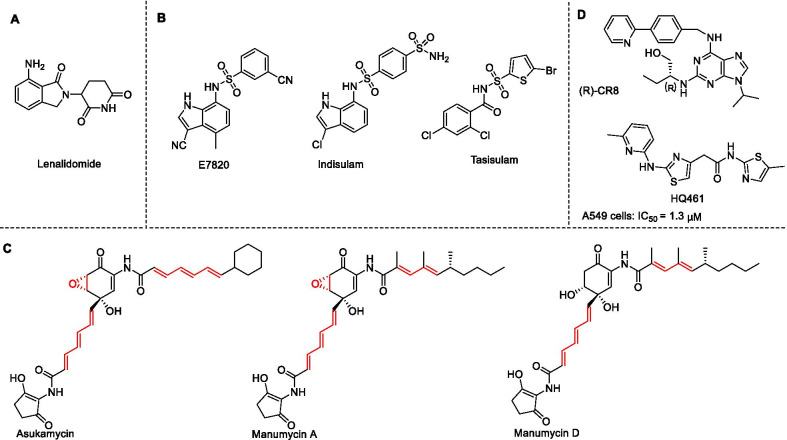
**a** Chemical structures of lenalidomide. **b** Chemical structures of E7820, indisulam, and tasisulam. **c** The structures of several Manumycin polyketide family members. Potentially reactive sites are shown in red. **d** Chemical structures of (*R*)-CR8 and HQ461

Molecular glue is a simple small molecule that can bind to the E3 ligase and the substrate protein simultaneously. Molecular glue can induce target proteins to undergo ubiquitin modification and degradation through the proteasome pathway [133]. A significant advantage is that molecular glue circumvents the limitations of traditional inhibitors, such as making some of the targets from "undruggable" to "druggable" [7]. At present, molecular glues of four E3 ligases (including CRBN, DCAF15, DDB1, and UBR7) have been identified. Krönke et al. reported the first molecular glue lenalidomide (thalidomide analog) (Fig. 17a) that induces the degradation of IKZF1/3 through CUL4/CRBN [134]. Faust et al. reported that arylsulfonamides (such as E7820, indisulam, and tasisulam (Fig. 17b)) act as molecular glues to bind to cullin RING ligase substrate receptor DCAF15 and splicing factor RBM39, promoting the degradation of the latter one in a proteasome-dependent manner. Notably, RBM39 was potently degraded in cells at 500 nM by E7820 while this compound has a relatively weak affinity for DCAF15, demonstrating that aryl sulfonamide selectively binds to and degrades RBM39 without the requirement for high binding-affinity ligands [135]. In addition, Isobe et al. showed that the members of the manumycin family of polyketides (Fig. 17c) are covalently linked to the cysteine ​​residue (C374) in RING E3 ligase UBR7 and bind to the neosubstrate tumor-suppressor TP53 in breast cancer cells, thereby acting as a molecular glue to increase p53 transcriptional activation and induce cell death [136]. Recently, several CDK inhibitors (such as (*R*)-CR8 and HQ461 (Fig. 17d)) have been identified as molecular glues of DDB1 [137, 138]. (*R*)-CR8 induced the formation of a complex between CDK12-cyclin K and the CUL4 adaptor protein DDB1 and promoted the ubiquitination and degradation of cyclin K [137]. Although the molecular glue may have good pharmacokinetic properties (such as oral bioavailability) due to lower molecular weight, very few molecular glue degraders have been discovered so far due to a lack of a systematic discovery and design strategy. Generally, molecular glue provides an attractive method for advancing the field of TPD, although the discovery process of molecular glue has a great contingency.

### Other monomeric degraders

**Fig. 18:**
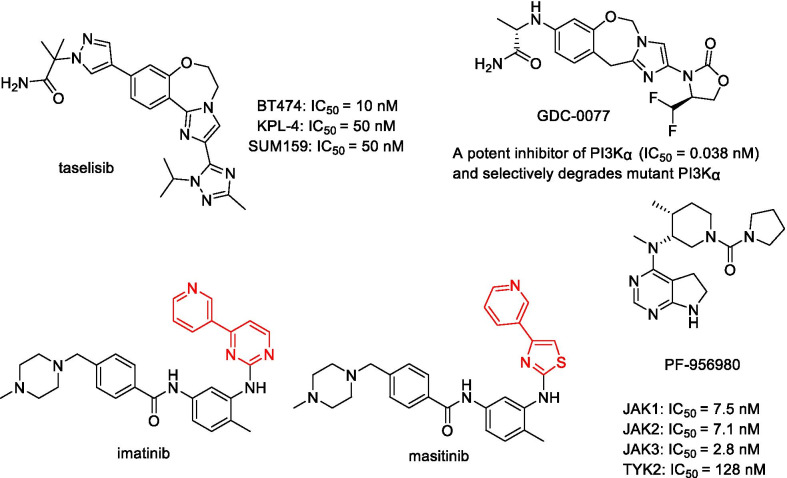
Chemical structures of taselisib, GDC-0077, imatinib, masitinib, and PF-956980. Taselisib and GDC-0077 can induce the selective degradation of mutant p110α. Imatinib and masitinib can induce the downregulation of wild-type c-Kit at 2 μM concentration

Some inhibitors have also been reported as monomeric degraders (Fig. 18). PI3Kα inhibitors taselisib and GDC-0077 can induce a selective degradation of mutant p110α in a proteasome-dependent manner [139, 140]. In addition to the proteasome pathway, some monomeric degraders exert their activity by other pathways. The c-Kit inhibitors imatinib and masitinib caused the downregulation of wild-type c-Kit at a concentration of 2 μM. However, the degradation of c-Kit was rescued by a co-treatment with methylamine but not with proteasome inhibitors, which indicates that the c-Kit degradation depends on a lysosomal mechanism [141]. However, there are still monomeric degraders whose mechanism is not yet clear. For example, PF-956980 is an ATP-competitive, reversible pan-JAK inhibitor, but it can cause significant JAK2/3 depletion, regardless of the presence or absence of the proteasome inhibitor. Therefore, it is difficult to determine whether this depletion depends on the proteasome [142].

Compared with heterobifunctional molecules, monomeric degraders have a relatively small molecular weight and are easier to penetrate the blood–brain barrier. They undoubtedly provide an alternative strategy to degrade target proteins. Currently, some monomeric degraders have entered clinical trials and gained market approval in the field of cancer treatment [143], such as Fulvestrant [94]. Although most of these monomeric degraders are accidentally discovered through empirical methods and have great risks and uncertainties [144], their ideal drug properties will encourage researchers to identify more monomeric degraders with better therapeutic effects.

## Conclusions and perspectives

Although the traditional SMIs are an important strategy for cancer treatment, they may face many challenges, such as drug resistance. The emergence of new degradation technologies represented by PROTAC can overcome the limitations of SMIs. Many emerging degradation technologies (such as LYTAC, AUTAC, ATTEC, and RIBOTAC) have also expanded the scope of degrading the disease-related targets, including undruggable targets (such as transcription factors, scaffold proteins, and RNA), providing more feasible strategies for the clinical treatment of related diseases. Although they are in their infancy and may have some critical issues that need to be resolved, they pointed the main direction of targeted therapy in the future.

## Data Availability

Not applicable.

## Abbreviations

AML: Acute myeloid leukemia
AR: Androgen receptor
ASGPR: Asialoglycoprotein receptor
ASOs: Antisense oligonucleotides
BCR: B cell receptor
BET: Bromodomain and extraterminal
BL: Burkitt lymphoma
BTK: Bruton’s tyrosine kinase
CDK: Cyclin-dependent kinase
cIAP1: Cellular inhibitor of apoptosis protein 1
CI-M6PR: Cation-independent M6P receptor
c-Kit: Stem cell factor receptor
CLL: Chronic lymphocytic leukemia
CRPC: Castration-resistant prostate cancer
Ctx: Cetuximab
DHFR: Dihydrofolate reductase
EGFR: Epidermal growth factor receptor
ER-α: Estrogen receptor-α
ESCC: Esophageal squamous cell carcinoma
EZH2: Enhancer of zeste homolog 2
FAK: Focal adhesion kinase
GAS: Group A streptococci
HD: Huntington's disease
HyT: Hydrophobic tagging
LRRK2: Leucine-rich repeat kinase2
M6P: Mannose 6 phosphate
MeBS: Bestatin methyl-ester
mHTT: Mutated huntingtin
MM: Multiple myeloma
ncRNA: Non-coding RNA
OVCAR: Ovarian cancer cells
PD-1/PD-L1: Programmed cell death protein 1/programmed cell death ligand 1
PEG: Polyethylene glycol
PI3K: Phosphoinositide 3-kinase
POI: Protein of interests
RNase: Ribonuclease
siRNA: Small interfering RNA
SMIs: Small-molecule inhibitors
TNBC: Triple negative breast cancer
TPD: Target protein degradation
UPS: Ubiquitin proteasome system
XIAP: X-linked inhibitor of apoptosis protein
